# The Evolving Role of Radioembolization in the Treatment of Neuroendocrine Liver Metastases

**DOI:** 10.3390/cancers14143415

**Published:** 2022-07-14

**Authors:** Khalil Ramdhani, Arthur J. A. T. Braat

**Affiliations:** Department Radiology and Nuclear Medicine, University Medical Center Utrecht, 3508 GA Utrecht, The Netherlands; k.ramdhani@umcutrecht.nl

**Keywords:** NEN, radioembolization, SIRT, neuroendocrine tumor

## Abstract

**Simple Summary:**

This review provides basic insights into radioembolization, also known as selective internal radiation therapy, in patients suffering from neuroendocrine liver metastases. Radioembolization is a treatment that uses radioactive beads that are implanted intra-arterially to locally irradiate liver tumors. The available literature on radioembolization in neuroendocrine liver metastases show promising results in terms of efficacy and toxicity and will be discussed in more detail. However, data in the field of NELM need clarification, and this review also discusses the caveats, challenges and new insights when considering radioembolization in neuroendocrine liver metastases.

**Abstract:**

At diagnosis, 21–50% of neuroendocrine tumors already have distant metastases, of which the liver is most commonly affected. Unfortunately, the presence of neuroendocrine liver metastases (NELM) is the most incriminating factor for survival. At NELM diagnosis, 60–70% of patients suffer from bilobar multifocal disease, making them ineligible for surgical resection. With limited systemic options, a clinical need for liver-directed treatments exists. Trans-arterial (bland) embolization, chemoembolization and radioembolization have been increasingly used in the treatment of NELM. In recent years, radioembolization (also known as selective internal radiation therapy) has gained attention due to promising tumor reductive results, limited toxicities and increasing scientific evidence. This review provides basic insights into radioembolization as a technique, a summary of available literature on radioembolization in NELM, and discusses caveats, challenges and new insights when considering radioembolization in NELM.

## 1. Introduction

Neuroendocrine neoplasms (NEN) consist of a very heterogenous group of tumors, contributing to large differences in patients’ disease burden, symptomatology, clinical and objective responses to different treatments, and prognosis. In accordance with the most recent WHO/ENETS criteria, tumor grading is the most common denominator for survival: grade 1 and 2 neuroendocrine tumors (G1-/G2NET) are regarded as well- to moderately differentiated tumors with a low Ki67 index (<3% and 3–20%, respectively); grade 3 NET (G3NET) as well- or moderately differentiated NET with high Ki67 index >20%; and neuroendocrine carcinomas (NEC) as poorly differentiated and with highly proliferative tumors (Ki67 index is most commonly >55%) [1,2,3]. However, within its heterogeneity, a well-established negative factor for survival for NEN patients is the presence of neuroendocrine liver metastases (NELM) [4]. Unfortunately, at diagnosis, 21% of all G1NET, 30% of all G2NET and 50% of all G3NET already have distant metastases, of which the liver is the most commonly affected [5,6]. In the presence of NELM, Frilling et al. provided an easy method to categorize liver involvement into three groups, based on tumor distribution in the liver [7]: from a ‘simple pattern’ (NELM involves 1–2 liver segments) to a ‘complex pattern’ (extensive unilobar disease with limited disease in the contralateral lobe) to a ‘diffuse pattern’ (bilobar or miliary disease). Whereas these ‘simple’ and ‘complex’ patterns allow surgical resection, the ‘diffuse’ pattern does not. Unfortunately, 60–70% of patients with NELM reside in the ‘diffuse pattern’ group, illustrating the clinical need for liver-directed treatments in light of the limited systemic options for NENs.

Liver-directed treatments for NELM can be divided into two categories: ablative localized treatments, e.g., radiofrequency ablation (RFA) or microwave ablation (MWA); or trans-arterial treatments, e.g., trans-arterial (bland) embolization (TAE), trans-arterial chemoembolization (TACE) and trans-arterial radioembolization (TARE). The latter technique is also known as selective internal radiation therapy (SIRT). Radioembolization is a more commonly used and simplified term but also a misnomer. Contrary to TAE and TACE, the primary effect is not to embolize vasculature and induce ischemia but to deliver high doses of radiation to tumor tissue via trans-arterial implantation. For simplicity, in this review, we will adhere to the term radioembolization. Where the localized ablative treatments can be used as an alternative to or in combination with surgical resection in the ‘simple’ and ‘complex’ disease patterns, the trans-arterial treatments are mainly applied in diffuse NELM [8,9]. 

Within trans-arterial treatments for NELM, radioembolization has gained a lot of attention over the last decade and reports high tumor objective response rates and limited toxicities [10]. As illustrated by the European Neuroendocrine Tumor Society (ENETS) guideline from 2016 and the European Society for Medical Oncology (ESMO) guideline from 2020, the role of radioembolization has extended, including early application as a tumor debulking treatment or as a salvage treatment in selected cases, after the failure of systemic treatments [4,11]. As NEN and NELM development are highly variable between individuals, the application of radioembolization needs to be determined on a case-by-case basis through discussions by multidisciplinary tumor boards (MDT). 

This review provides insights into radioembolization as a technique, a summary of available literature on radioembolization in NELM, and a discussion of caveats, challenges and new insights when considering radioembolization in NELM.

## 2. How Is Radioembolization Performed?

Pre-radioembolization work-up is quite similar to other (minimally) invasive treatments, including clinical assessment, laboratory testing and imaging investigations. The minimal requirements and additional assessments that could be considered during the work-up for NELM are depicted in Table 1.

**Figure 1:**
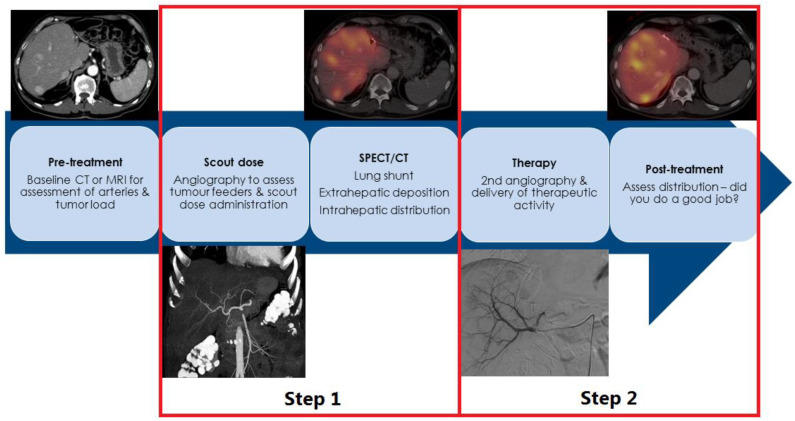
Graphical representation of a radioembolization treatment.

Radioembolization is a multidisciplinary treatment (involving an interventional radiologist and nuclear medicine physician) and always consists of a two-step approach (Figure 1). Firstly, a treatment simulation is performed, also known as a scout-procedure, with the administration of scout particles, either technetium-99 m macroaggregated albumin (^99 m^Tc-MAA) or a small amount of holmium-166 microspheres (^166^Ho-scout dose, QuiremScout^®^, Quirem Medical, Deventer, the Netherlands), followed by imaging. Secondly, the actual radioembolization procedure with the administration of the therapeutic activity of particles can be used with one of three commercially available particles: yttrium-90 (^90^Y)-loaded glass (Theraspheres^®^, Boston Scientific, Marlborough, MA, USA), resin microspheres (SirSpheres^®^, SIRTex medical, Woburn, MA, USA), or ^166^Ho-microspheres (QuiremSpheres^®^, Quirem Medical, Deventer, the Netherlands). Thereafter, clinical, laboratory and imaging evaluation is performed for at least 6 months [9].

Prior to the first step (scout angiography), an arterial-phase contrast-enhanced CT (CECT) gives the interventional radiologist insights into the arterial variants or collaterals, which has been shown to limit procedure time and radiation exposure to the patient and personnel [12,13]. The angiography can be performed using a femoral or radial approach, and liver and tumor vasculature is assessed using digital subtraction angiography. Following the angiography assessment, additional periprocedural cone beam CTs (CBCT) are recommended as they allow the interventional radiologist to confirm total tumor coverage during the procedure and detect potential arteries supplying extrahepatic tissues, so-called culprit vessels [8]. Culprit vessels should be avoided through a more distal placement of a microcatheter beyond the origin or by coil embolization of the culprit vessel to avoid any extrahepatic deposition of radioactivity during the actual treatment. A distal placement of the microcatheter beyond the origin of a potential culprit vessel is preferred (‘limiting your footprint’); the coil embolization of culprit vessels may induce the formation of small collaterals, increasing the difficulty of treatment or causing an absolute contra-indication (inevitable extrahepatic deposition of activity) [14]. Once the interventional radiologist has determined a safe injection position(s), the scout particles are administered. 

Subsequently, after the closure of the femoral or radial access, patients are taken to the nuclear medicine department for SPECT/CT imaging to exclude ‘lung shunting’ (the shunting of particles via physiological or tumor-induced arteriovenous shunts) and extrahepatic depositions of activity in the abdomen (via culprit vessels). In the event that large amounts of particles shunt towards the lungs, this is a contra-indication for treatment as this may induce (fatal) radiation pneumonitis 2–8 weeks following treatment [15]. In the case of extrahepatic depositions of activity in the gastrointestinal tract or pancreas, patients could develop radiation-induced ulcers or pancreatitis, which are difficult to treat. After excluding lung shunting and extrahepatic depositions, patients are often scheduled for the treatment procedure within 1–3 weeks [8].

Following the actual radioembolization procedure, the post-treatment imaging of the particles themselves should be performed, either with ^90^Y-PET/CT (or ^90^Y-Brehmstralung SPECT/CT) or ^166^Ho-SPECT/CT, to confirm the intrahepatic distribution and the absence of extrahepatic depositions of activity (Figure 1) [8]. 

To date, no evidence is available on the use of prophylactic medication in NELM. Based on clinical experience, standard octreotide infusion is not recommended, except in patients experiencing several carcinoid syndromes [9]. Prophylactic antibiotics should be considered in patients with a bilidigestive anastomosis but not in the general population [16]. Prophylactic proton pump inhibitors, anti-emetics and dexamethasone were commonly used at the start of radioembolization, but most experienced centers refrain from using any prophylactic medications [17].

## 3. SIRT in NEN: Salvage Setting

To date, many groups have collaborated to define the role of radioembolization in the treatment of NELM. The main body of evidence used to reside with many small retrospective studies consisting of mixed populations, of which the majority was in a salvage, late-stage setting. In the past 5 years, several larger retrospective studies and registries have been published further evaluating the role of radioembolization in NELM. Although presented in mixed populations, the results are very promising. Table 2 summarizes the most important scientific evidence available to date for salvage radioembolization. 

**Figure 2:**
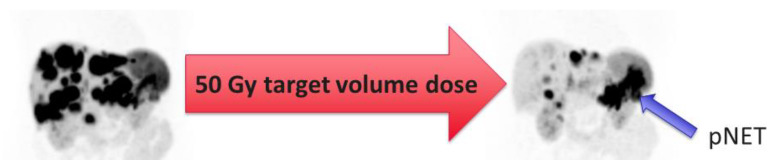
Radioembolization in a patient with an irresectable grade 1 pNET/insulinoma, suffering from frequent hypoglycemic crises (even with continuous enteral feeding and medication) after failure of somatostatin analogs (SSA), chemotherapy with capecitabine + temozolomide (CAPTEM) and peptide receptor radionuclide therapy (PRRT). Left: Pretreatment ^68^Ga-DOTATOC PET/CT depicting the pNET and liver metastases. Treated with a whole liver radioembolization in a single session, with ^90^Y resin microspheres (monocompartment modelling, 50 Gy target volume dose). Within two weeks, this clinically resulted in a significant reduction in insulin production, allowing the cessation of enteral feeding and a dose reduction in supportive medication. Right: Post-treatment ^68^Ga-DOTATOC PET/CT 6 months after treatment showing evident tumor reduction. Although clinically stable for a long time, follow-up imaging showed a minor progression of disease after 3.2 years.

Interestingly, together, these studies report over 1200 patients with quite similar findings, illustrating some degree of robustness of data and confirming the safety and efficacy of the radioembolization of NELM in a salvage setting (Table 2 and Figure 2). In addition to similar results for objective endpoints (imaging-based response and overall survival), all studies show a positive correlation with low tumor grading, obtaining DCR at first evaluation and limited intrahepatic tumor burden with OS. Several differences can also be noticed: the most interesting was the influence of the presence of extrahepatic disease. Braat et al. found a significant negative correlation with OS, whilst Schaarschmidt et al. and Wong et al. did not. However, the majority of the patients the study of Braat et al. had more extensive extrahepatic disease (66%). To date, only Braat et al. reported clinical outcomes, indicating the reduction in or resolution of hormone-related complaints in 44% and 34% of patients, respectively [9,20,21].

## 4. Radioembolization in Earlier Lines or Combinations Treatments

**Figure 3:**
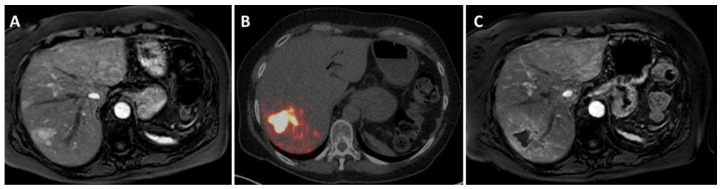
Radioembolization in a patient with oligoprogression of a liver metastases in liver segment 7 (**A**), previously treated with 4 cycles of PRRT after a Whipple resection of the primary non-functional pNET. No disease elsewhere on ^68^Ga-DOTATOC PET/CT. Gadolinium-enhanced MRI shows a larger lesion with multiple satellite lesions in its vicinity (with diffusion restriction). Surgical resection and ablative techniques were considered unsuitable, thus, MDT decided on a selective ablative radioembolization. (**B**) Post-treatment ^90^Y PET/CT (300 Gy target volume dose in segment 7), revealing a high accumulation of particles in all metastases. (**C**) MRI 3 months after treatment showing complete tumor necrosis of all metastases and some radiation-induced changes in the surrounding healthy liver tissue.

As the liver is the most commonly affected organ for NEN metastases, independent of tumor origin and often the only affected organ after the resection of a primary tumor, the application of radioembolization in an earlier line of the disease is increasingly debated. As shown in the current ENETS and ESMO guidelines, liver-directed treatments should be considered in specific cases [4,11]. To date, only one retrospective study specifically investigated the role of radioembolization in a second-line setting (following surgical resections and first-line somatostatin analogs, as shown in Figure 3). Schaarschmidt et al. showed a median hepatic PFS of 18.6 months and median global PFS of 18.8 months, which is slightly better than the results obtained in a salvage setting (Table 2). Logically, prolonged median OS was found (44.8 vs. 30.6 months) in the group treated in the second-line compared to the salvage setting group [20]. 

The mainstay in the treatment of metastatic disease resides in the use of systemic treatments, most commonly somatostatin analogs (SSA), peptide receptor radionuclide therapy (PRRT) and chemotherapy [4,11]. In patients with significant intrahepatic tumor burden or aggressive disease, systemic treatments tend to have less prolonged effects [22,23]. Combining a systemic and/or targeted treatment with a liver-directed treatment seems logical to boost the benefit for patients suffering from high intrahepatic tumor burden or patients with mainly NELM (liver-only or so-called “liver dominant disease”). 

In previously described retrospective studies, many patients received (high-dose) SSA simultaneously with radioembolization, either for symptom control or potentially to prolong the tumor reductive effect of radioembolization. Unlike other treatments, e.g., first-line chemotherapy, no firm scientific evidence suggests a synergistic effect of SSA, and this was not separately analyzed in the aforementioned studies [24,25]. Presently, only one small study is being conducted looking at this specific combination (NCT02859064). 

Currently, three smaller studies in grade 1 and 2 NETs are being conducted to combine radioembolization with systemic treatments (Table 3). 

The study by Soulen et al. was the first in which ^90^Y resin radioembolization was combined with systemic chemotherapy capecitabin + temozolomide (CAPTEM) [26]. In this study, 21 patients with NELM of different origins were analyzed, of which the majority were treated in a second-line setting. The authors’ report showed high response rates and long survival and suggested a synergistic effect, mainly to the benefit of pNET patients. Hepatic failure due to radioembolization-induced liver disease (REILD) was encountered in one patient. To date, no follow-up data have been reported, but a follow-up study has been initiated (NCT04339036).

Kim et al. rapidly followed this with a second study combining everolimus and pasitreotide, based on an earlier study, and adding ^90^Y resin radioembolization [27]. This was a phase 1b study, in which the dose of everolimus was escalated, whilst the pasitreotide and radioembolization were standardized. In line with the findings of the previous RADIANT trials, a dose of 10 mg of everolimus was safe and effective, even in combination with pasitreotide and radioembolization. No additional hepatotoxicity was noticed. To date, no follow-up data have been presented, and to the knowledge of the authors, no follow-up study has been initiated.

**Figure 4:**
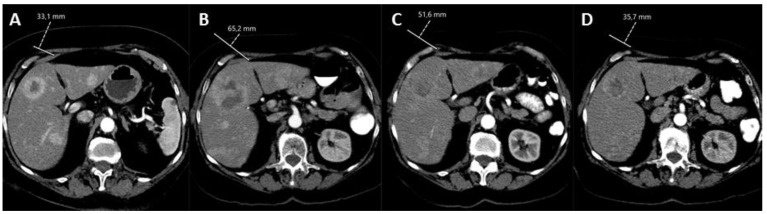
Patient with a grade 1 pNET with liver metastases, who participated in the HEPAR PLuS trial. (**A**) Baseline CT prior to PRRT with 4 cycles of 7.4 GBq ^177^Lu-DOTATATE. (**B**) CT 3 months after PRRT depicting evident progressive intrahepatic disease. (**C**) CT 3 months after additional ^166^Ho-radioembolization showing increased necrosis, size reduction and reduced enhancement of neuroendocrine liver metastases, and stable disease according to RECIST 1.1 (−22%). (**D**) CT 12 months after additional ^166^Ho-radioembolization showing advancing partial response according to RECIST 1.1 (−44%) and tumor reduction.

The most recent study by Braat et al. (“HEPAR PLuS”) combined PRRT with ^166^Ho-radioembolization, by adding radioembolization within 20 weeks after the fourth cycle of PRRT [28]. The authors concluded that the combination was safe and effective. REILD was encountered in one patient. However, due to the heterogeneity of the group and the selection bias introduced by patient inclusion after the completion of PRRT, a patient population with a poor prognosis was selected. Five out of thirty-one (17%) of the included patients already failed PRRT (with only intrahepatic progressive disease). Nonetheless, high ORR, both RECIST 1.1 and mRECIST, and durable responses during the first year in follow-up were reported, thus the combination seems promising, and no loss in quality of life was reported (Figure 4).

Follow-up data on laboratory markers and survival were recently reported [29]. Post-PRRT bone marrow depression can be clinically challenging, but the follow-up data show that it seems safe to add radioembolization to PRRT in the absence of any hematotoxicity without significantly prolonging hematological recovery [29]. 

As illustrated, the data are limited; nonetheless, the data show that combining radioembolization with systemic treatments is promising and safe. The studies by Braat et al. and Frilling et al. illustrate that, following radioembolization, progressive disease and PFS occurred through the progression of extrahepatic disease or non-treated liver volumes [28,30]. Implementing radioembolization in liver-only disease as a monotherapy makes sense but becomes more complicated with the presence of extrahepatic disease. Thus, combining systemic treatments with radioembolization will benefit both treatment modalities, and further investigations are warranted. 

## 5. Concerns, Limitations and Future Perspectives

In terms of short-term toxicity, the most feared complication is REILD, which is a form of sinusoid obstructive syndrome (SOS), caused by excessive radiation to healthy liver tissue [31]. However, the data presented in Table 2 show that REILD occurrence is incidental (0–1.8%). In patients with a history of Whipple resection and bilidigestive anastomosis, trans-arterial treatments may induce biliary ischemia (as the biliary tree is vascularized via the artery), and subsequently, may become infected by gastrointestinal bacteria in the absence of a physical barrier and retrograde colonization [8]. Especially following TACE, this is a very common complication [16]. However, liver abscesses following radioembolization in the presence of a bilidigestive anastomosis (with or without antibiotic prophylaxis) are limited. Theoretically, this seems logical as, with radioembolization, inducing ischemia is not the goal (misnomer), and the amount of biliary ischemia is significantly less pronounced than with TAE or TACE. Therefore, in patients with a bilidigestive anastomosis eligible for an embolization therapy, radioembolization is recommended, and TAE or TACE is discouraged. Other short-term complications (radiation pneumonitis <1%, gastrointestinal ulcers <2% or radiation-induced pancreatitis ~0%) are very uncommon in NEN [9].

Long-term toxicity, and in particular, long hepatotoxicity is an area of scientific debate has a lack of proper evidence [32,33,34]. Mainly in North America, concerns were raised due to suggested long-term hepatotoxicity, showing as cirrhosis-like morphology on imaging studies without clinical complaints. This was initially encountered in patients treated with radioembolization in the pre-PRRT era, who subsequently received PRRT in later disease stages [35]. Subsequent small, retrospective studies suggested that mainly patients treated with whole liver radioembolization were prone to developing long-term hepatotoxicity [36,37]. Unfortunately, these small studies could not be confirmed in the larger retrospective studies or in the prospective study by Braat et al. [9,28]. In a recent single center study, long-term hepatotoxicity was compared between patients treated with TACE and radioembolization, and no significant difference was encountered (22% vs. 29%, respectively) [32]. One may wonder whether this ‘problem’ is truly a concern. Additional issues in this scientific discussion reside in the confusion caused by the variable definitions of ‘long-term hepatotoxicity’, the discussion of confounded data (as most patients received subsequent treatments during follow-up), and the absence of one of the most important factors in radioembolization, namely in vivo particle/radiation distribution assessment, called ‘dosimetry’. 

Unfortunately, a lack of dosimetry is the largest caveat of all the studies on radioembolization in NEN. As radioembolization is a two-step approach (Figure 1), physicians can use the scout procedure SPECT to predict the intrahepatic distribution of particles prior to the actual treatment. Dosimetry that is based on scout procedure SPECT allows dose escalation without increasing the risk of complications. In most centers, hepatocellular carcinoma is the number one indicator of radioembolization, and in this regard, indisputable evidence has been generated, emphasizing the importance of dosimetry [38,39,40]. In the landmark study, DOSISPHERE-1, patients treated with ^90^Y glass microspheres by a ‘one-size-fits-all’ approach (monocompartment model; control group) had significantly poorer results, compared to the patients that were treated in a patient-personalized fashion (multicompartment modelling), aiming for a minimum tumor-absorbed dose of 205 Gy. With multicompartment modelling, ORR increased to 71% (vs. 36%). PFS was prolonged to 6.0 months (vs. 3.4 months), and in properly dosed patients, OS was significantly prolonged to 26.6 months (vs. 7.1 months) [38]. 

To date, only one study evaluated dosimetry in NEN. Ebbers et al. analyzed 26 patients (128 tumors) treated with ^90^Y glass radioembolization in a single center. A clear dose–response relationship, independent of NEN grade, was confirmed [41]. A minimum tumor-absorbed dose of 150 Gy, and preferably more than 200 Gy, significantly increases the likelihood of receiving an RECIST 1.1-based ORR (>80%). Due to the hypervascular nature of NEN, the encountered healthy liver tissues dose was very low, limiting the possibility of establishing a dose–toxicity relationship [42]. A dose–survival relationship could be established (tumor dose >150 Gy); however, when correcting for the intrahepatic tumor burden, this relationship became non-significant. Although this was a small study, it illustrates that patient-personalized treatment based on dosimetric assessment is the way forward, in line with the developments in hepatocellular carcinoma [41].

Whereas dosimetric assessments used to be performed with in-house developed software, currently, all manufacturers of commercially available therapeutic microspheres acknowledge the importance of dosimetry, and multiple dedicated software packages are commercially available. Therefore, the application of multicompartment modelling will rapidly become standard practice. 

## 6. Conclusions

Hepatic radioembolization is safe and effective as a monotreatment in NEN. Based on current evidence, the exact application of radioembolization in NEN care remains unknown, and the scientific debate on suggested long-term toxicities remains unresolved. The application of radioembolization should be considered on a case-by-case basis through multidisciplinary discussion. Upcoming clinical and technical developments in the field will ensure a more promising role for radioembolization in NEN care.

## Figures and Tables

**Table 1:** Pre-radioembolization work-up.

Clinical Assessment	Laboratory Testing	Imaging Work-Up
**Minimal**		
ECOG performance score	Bilirubin, ALP, AST, ALT, albumin	gdMRI/CECT for intrahepatic tumor load ^1^
Signs of hepatic dysfunction (Child–Pugh score)	Creatinine, eGFR	Early-phase CECT for arterial vasculature
NET hormone-related symptoms	Tumor markers (e.g., CgA, gastrin)	
**Additional**		
In selected cases, Fibroscan or gastroscopy to assess esophageal varices	Hb, hematocrit, WBC, platelets	SSTR-PET/CT for total body tumor load ^1^
	Coagulation (e.g., Prothrombin time or INR)	FDG-PET/CT for tumor grade distinction, excluding aggressive disease.

Legend: ECOG = Eastern Cooperative Oncology Group, NET = neuroendocrine tumor, eGFR = estimated glomerular filtration rate; ALP = alkaline phosphatase, AST = aspartate aminotransferase, ALT = alanine aminotransferase, CgA = chromogranin A, Hb = hemoglobin, WBC = white blood cell count, INR = international normalized ratio, gdMRI = gadolinium-enhanced magnetic resonance imaging, CECT = contrast-enhanced computed tomography, SSTR = somatostatin receptor; PET/CT = positron emission tomography/computed tomography, FDG = fluorodeoxyglucose. ^1^ tumor load = fractional tumor involvement.

**Table 2:** Landmark papers on salvage radioembolization in NEN.

	Year	N	ORR *	DCR *	PFS	OS	REILD
			%	%	Months	Months	n (%)
Devcic et al. ^†^ [10]	2014	435	50	86	NR	28.5	NR
Peker et al. [18]	2015	38	46	83	NR	39	0
Barbier et al. [19]	2016	54	54	94	NR	34.8	1 (1.8)
Braat et al. [9]	2019	244	16	91	NR	31	2 (0.8)
			43	91			
Schaarschmidt et al. [20]	2022	297	41.3	83.5	15.9	30.6	2 (0.8)
Wong et al. [21]	2022	170	36	69	25	33	1 (0.6)

Legend: NR = not reported, n = number of procedures, ORR = objective response rate, defined as complete + partial response, DCR = disease control rate, defined as ORR + stable disease, PFS = median or mean progression free survival, OS = median or mean overall survival, REILD = radioembolization-induced liver disease. * Response within or at 3 months according to RECIST 1.1 in regular font and in italics according to mRECIST. ^†^ Only meta-analyses on data before 2014, and other studies presented are original articles.

**Table 3:** Combination treatments with radioembolization in NEN.

Author	Year	n	Population	Procedures	ORR *	PFS ^†^	OS
Soulen et al. [26]	2018	21	Grade 2 NELM	capecitabin 600 mg/m^2^ twice daily for 14 days temozolomide 150 to 200 mg/m^2^ in divided on days 10 to 14. ^90^Y resin radioembolization 7th day of cycle 2	74%	NR	NR
Kim et al. [27]	2018	13	Grade 1 + 2 NELM	3 + 3 dose escalation of everolimus 2,5–5–10 mg Pasitreotide 600 µg twice daily ^90^Y resin radioembolization day 9 and 37	46%	18.6	46.3
Braat et al. [28]	2020	31	Grade 1 + 2 NELM	Standard 4 cycles of 7.4 GBq ^177^Lu-PRRT ^166^Ho-radioembolization <20 weeks after 4th PRRT	43%	30.1	40.8

Legend: n = number of patients, ORR = objective response rate, defined as complete + partial response according to RECIST 1.1, PFS = median progression-free survival, PRRT = peptide receptor radionuclide therapy with lutetium-177-DOTATATE, NR = not reached. * Intrahepatic ORR. ^†^ Progression-free survival in months.
